# Pathology and Practice of Medicine

**Published:** 1861-01

**Authors:** Richard J. Dunglison

**Affiliations:** One of the Physicians to the Pennsylvania Institution for the Instruction of the Blind; Honorary Local Secretary to the Sydenham Society of London, etc.


### PATHOLOGY AND PRACTICE OF MEDICINE.

BY RICHARD J. DUNGLISON, M.D.,

ONE OF THE PHYSICIANS TO THE PENNSYLVANIA INSTITUTION FOR THE INSTRUCTION OF THE BLIND; HONORARY LOCAL SECRETARY TO THE SYDENHAM SOCIETY OF LONDON, ETC.

I.—*Remarks on Heat-Apoplexy or Sun-Stroke, as observed at Lucknow, in the Hospital of the 1st European Bengal Fusiliers, during the months of May and June,* 1859. By JAMES P. BROUGHAM, M.D., Surgeon to the Presidency General Hospital. (Glasgow Med. Journal, Oct. 1860, p. 372.)

Occasionally in the public services we find medical officers who are willing and anxious to record their observations for the good of the profession; and opportunities are often afforded to them for observing morbid phenomena, which are not available to the mass of the public. Thus, from India, where such rich material is at hand, in regard to the influence of solar heat as a cause of disease, we naturally expect to have much information upon insolation afforded us, and this contribution of Dr. Brougham is a valuable addition to the literature of the subject.

After detailing the symptoms of this disease, such as diminished sensibility, nervous twitchings of the facial muscles, increased heat of surface, insensibility of the pupil to light, involuntary or semi-conscious movements, Dr. Brougham proceeds to state that in the post-mortem examinations made in almost every case of death which occurred in the Fusilier Hospital the following appearances were presented. His observations are of value from his experience and position, and are entitled to credit and consideration.

First. There was invariably a very remarkable effusion of serum, both beneath the arachnoid and in the lateral ventricles, amounting, in some cases, to two ounces; this fluid was also found within the theca vertebralis. There was also much venous congestion about the base of the brain and medulla oblongata. In no case was there rupture of any vessel, or sanguineous apoplexy.

Secondly. The lungs externally were always free from inflammation, except where disease had previously existed; in some few cases a small quantity of serum was found within the pleural sac. The lung tissue, however, invariably seemed much redder than usual, and crepitated on pressure, while frothy mucus exuded abundantly from incisions made into the substance.

Thirdly. There was almost invariably a considerable quantity of serum within the pericardium, and in one instance fresh fibrin had been deposited between the pericardium and serous covering of the heart. The right side of the heart was always engorged with very dark, fluid blood, and the left was empty. No trace of internal disease of this organ was ever seen.

Fourthly. The liver seemed always to contain some excess of blood, but was healthy in structure.

Fifthly. In one case the spleen was enlarged, and was engorged to a considerable extent.

All other organs were in a normal state.

It is impossible to remember the common track of the fourth, fifth, and eighth nerves, as pointed out by Sir C. Bell, and not be struck with the fact of the twitchings of the facial muscles, the working of the fingers about the throat, the difficulty of swallowing, and the accumulation of mucus within the bronchi, the almost inflamed state of the lungs, and not think there is a track of nerve matter the actions of which have become abnormal, irregular, or suspended.

Is the affection of the nerves primary, depending on the solar rays acting on the brain, or secondary, depending on a deranged state of the pulmonary function?

The nerves supplying the lungs are not alone affected; there is entire loss of voluntary motion, or nearly so, and sensation, as he has already stated, has almost gone; the entire nervous system, therefore, suffers. It is certain, he thinks, that this does not depend on the direct rays of the sun, many men who suffered not, having been exposed, others, again, having been seized during the hours of darkness, early in the morning. All, however, suffered alike, with the same moaning or expiration, same want of inspiration, same difficulty of swallowing. The pupil was also fixed, contracted, or dilated, in either case insensible, the heart beating sensibly up to, or beyond the period of death.

Exposure to heat tends to produce venous turgescence, and disturbs the balance of the circulation, partly by its mere physical effect in causing general expansion; but there are other causes which certainly act very differently. The action of the heart is invariably quickened, but with this is marked depression of the animal functions, as shown by listlessness and want of energy, and should the temperature increase, or even remain undiminished, we may expect these effects to become exaggerated, particularly when those exposed have been weakened by previous illness, or are called on to make any violent or continuous effort.

The writer looks on the attack not as the commencement, but as the end of insidious blood poisoning; and though it is then that the lungs begin to suffer more directly through the brain, it is because that organ has been supplied with blood not fitted for nutrition, so that it becomes embarrassed in its functions, or these are entirely suspended; in either case the impression produced is apt to increase and proceed to a fatal termination.

II.—*On the Non-Prevalence of Pulmonary Consumption in the Hebrides, and along the Northwestern Coast of Scotland.* By JOHN E. MORGAN, B.A., Oxon. (Brit. and For. Med.-Chir. Review, October, 1860, p. 483.)

The result of inquiries made personally and by letter, in the region of country extending between the island of Mull in the south, to the whole of the Hebrides, and extreme portions of the northwestern coast of Scotland, is detailed by Mr. Morgan to exhibit the rarity of tubercular consumption in those localities. The replies elicited from physicians settled there testify with singular unanimity to the same fact. The conditions under which the people lived, their modes of living, their dress and diet, and their domestic life, are all referred to, but even though some of the peculiar circumstances of that district of country are believed by the medical men residing there to account sufficiently for the exemption from phthisis, yet the observations of the writer do not confirm that view. He finds other countries similarly situated physically and in every way, but suffering far more from tubercular disease of the lungs.

Some of the points detailed in regard to the habits and occupations of the people are interesting, and worthy of being mentioned, because practitioners there consider them as influential causes of the non-prevalence of phthisis. They live in a mild, equable, moist climate, deficient in sunshine; their summers are cold enough for fires, and their winters are too warm for continued snow and ice; shrubs like the azalia japonica and fuchsias flourish in the open air in winter, if partially screened from the wind. The majority of the natives are fishermen, as well as cultivators of a small piece of land, and are naturally much exposed to the weather, sometimes passing a great part of the night at their trade. The articles worn are woolen externally, while the under-garments are cotton; and from May to October the women and children go barefooted.

They live on meal, potatoes, milk, and fish, and at certain seasons almost wholly on oat-cake and porridge; the first containing very little oleaginous matter, and not, therefore, preventing phthisis by supplying an oleaginous diet. The amount of ozone in the atmosphere is very great, but as that is a fact also observed always near the sea, whether the district of country be very subject to phthisis or but little so, it may not have much weight as a preventive cause.

The highlanders of the Hebrides are a mixed race, the Celtic element being very apparent, and there is no reason for believing race to have much influence. The huts or cottages in which they live are of very rude construction, yet the system of ventilation is very efficient. The scrofulous diathesis is frequently met with notwithstanding, yet not often accompanied by tubercular disease of the lungs.

It looks, therefore, as if some special cause of protection to the lungs existed, and Mr. Morgan thinks the use of peat fuel of a very good quality is sufficient, when inhaled in such quantities by the inmates, to act as a shield to the lungs. The smoke accumulates in the houses, and is breathed constantly. If the inhalation of different substances, and among them tar, be a valuable mode of treatment in cases of phthisis, the view here expressed would seem a plausible one, for the amount of tar in the peat is very considerable.

III.—*A Case of Leucocythæmia.* By DR. GEORGE SHEARER, Resident Physician, Royal Infirmary, Edinburgh. (Edin. Med. Journal, July, 1860, p. 48.)

A young man, aged twenty-four, a miller by trade, admitted to the infirmary under the care of Dr. Gairdner, affords an interesting illustration of leucocythæmia. Three weeks elapsed from his admission to the time of his death, and "the following is a summary of the facts of the case in regular sequence: Anæmia, languor, and debility; epistaxis; headache; bleeding from the gums; renal pain, with lithiasis; febrile symptoms; disappearance of lithic acid, and appearance of lithates and albumen; diarrhœa; reappearance of lithic acid; uncontrollable epistaxis; hæmatemesis; otitis; exhaustion and death."

The crystalline deposit in the urine, on the third or fourth day after admission, consisted mainly of hexagonal crystals of lithic acid, with a few of the ordinary rhomboidal crystals. These, we have already said, afterward disappeared. Post-mortem examination revealed leucocythæmia, enlarged spleen, fatty liver, petechiæ on the mucous membrane of stomach and on the serous surfaces of the pericardium and endocardium.

The case detailed by Dr. Shearer gives him a field for reflection, which he discusses in the following suggestions:—

1. Enlargement and activity of the spleen is not the only condition involving increase of the white corpuscles, there being at present a case in the infirmary in which this condition of the blood coexists with enlargement of the whole lymphatic system of glands, without any detectable enlargement of the splenic organ.

2. The fact of a great excess of white corpuscles in the blood in cases of leucocythæmia being accompanied by constant diminution of the red discs, appears to militate against the theory put forward by Wharton Jones, and supported by Bennett and others, that the latter are derived from the former by liberation of their included nuclei; for, according to their theory, increased activity in the formation of the white ought, *pari passu*, to be attended by increased development of red discs, while the reverse is the case. Comparative increase of the white corpuscles is seen in a variety of organic diseases, especially chest affections; but it also occurs in dysentery, diarrhœa, paraplegia, etc; in all of which one general condition was observed, viz., depreciation of the appetite, and emaciation. These facts, Dr. Shearer thinks, point to the blood itself as the primary source of origin of the red discs, and in the diseases mentioned there is either a deficiency of nutritive pabulum taken into the blood for the production of the red corpuscles, or these are rapidly melted down to supply the elements of the discharge. In leucocythæmia, again, the nutritive pabulum is appropriated for the formation of the white corpuscles, the blood being thereby impoverished to the extent to which these are increased; development of the red discs is consequently kept in abeyance, and anæmia is again the result.

3. The deficiency of color in the urine and the salts obtained from it depends probably upon the same cause as the pallor of the general surface, viz., deficiency of red globules and hæmatin in the blood.

4. Careful study of the deposit of lithic acid seemed to warrant the inference that the common or lozenge-shaped crystal is derived from the perfect hexagonal form by shortening of the lateral planes of the latter; but this does not explain the formation of the true rhombic crystal, which is an irregular form.

5. Hemorrhage from various mucous surfaces forms a prominent feature of this disease, and may depend partly upon the increased tension maintained in vessels by the absolute increase of volume in the mass of the blood, and partly upon the imperfect nutrition of the walls of the capillaries from the inferior quality of the blood for histogenetic purposes.

6. The white corpuscles, we know, are closely allied to fibrin in composition and character; fibrin is increased in febrile and inflammatory diseases, and accompanying this is an increased elimination of lithic acid, or lithates, by the kidneys. Can any relation exist between the lithuria present in this case, and the increase of white corpuscles in the blood?

IV.—*The Leprosy of the West Indies.* By R. SWEETING, M.D., etc. (Medical Times and Gazette, September 1, 1860, p. 208.)

We have here the result of the observations of a resident of the Bahamas, upon a disease which has elicited much discussion at various times, and especially with a view to the question of alleged identity with the leprosy of the ancients. The analysis of symptoms as detailed by Dr. Sweeting is as follows: General cachexy of the system, lasting for months or years perhaps, before any appearance of cutaneous disease, the features assuming a sickly and repulsive aspect, the face being slightly mottled, and the backs of the hands congested. These, and other symptoms which show it to be of constitutional origin, are followed by an affection of the skin, beginning about the lower lobe of the ear, with a pendulous enlargement of that structure. The face becomes covered with innumerable elevations of the skin, consisting of hypertrophy and induration of the true tissue of the dermis, throwing out a watery and semi-purulent secretion, which, incrusting, gives a dirty-white appearance to the surface, and the face becomes a mass of irregular elevations which gradually end in ulcerations.

The disease, so far as the skin is concerned, is almost limited to the hands and face. The majority of leprous patients die of phthisis. Leprosy is not a contagious disease, and no patient in the hospital has ever been attacked with it from association with cases suffering from that malady. The general opinion of its origin is, that it arises from the want of sufficient change in the food of the poor. Dr. Sweeting suggests that, like the bronchocele of Alpine valleys, it is in some way due to an imperfect dietary continued through generations under certain climatic conditions. In regard to treatment, where leprosy is fully developed, one treatment is about as good as another, and about as useless. Solutions of chlorate of potassa sometimes do good. In the incipient stage, relief is often obtained from a varied and generous diet, warm clothing, and from the remedies which experience has proved to be so useful in common lepra and psoriasis.

V.—*Contributions to the Pathology and Therapeutics of Typhus Fever. On the Identity of Typhus and Typhoid Fevers; community of Origin.* By JOSEPH BELL, M.D., one of the Physicians and Clinical Lecturers, Royal Infirmary, Glasgow. (Glasgow Med. Journal, October, 1860, p. 300.)

This paper is one of a series of valuable observations upon typhus fever, described by one who has had abundant opportunities for studying its pathological phenomena and lesions. After showing in the previous number of the journal that both forms of fever, the typhus and typhoid, were really identical in their symptoms, at least so far as the term identical is applicable to the symptoms of disease, and that the intestinal lesions in both forms of fever were essentially the same, differing merely in the degree of intensity and extent, he proceeds now to adduce evidence that the facts mentioned prove unquestionably that the two forms of fever have a common origin, and that the objections which have been brought forward against this conclusion are perfectly untenable, and founded on obvious fallacies, bold assumptions, and misrepresentations.

Dr. Bell supports the doctrine of the identity of typhoid and typhus fevers upon the threefold basis of the similarity of the symptoms during life, the essential resemblance of the intestinal lesions after death, and the community of origin. The last topic forms the subject of the present paper; we regret that its length will prevent us from giving more than a condensed synopsis of it.

Numerous instances of both forms of fever exist, occurring in the same family and at the same time and in the same bed, and the experience of Dr. Stokes* is appealed to, who says: "We have frequently observed, in cases where a large number of a single family have been successively attacked by contagious fever, that every form of the disease, from the most malignant typhus to the mildest typhoid fever, may be presented by different members of the family." A number of interesting cases are detailed by Dr. Bell in confirmation of this fact. The objection which the advocates of non-identity urge is, that these cases are rare; but this limitation of their experience does not disprove numerous observations of many other competent observers.

Then comes a discussion of the question of "foci of infection," and of the views of Jenner and Murchison. The former attempts to show that typhus and typhoid fever arise from different foci, that each has its own specific cause, or morbid poison, as essentially distinct as that of measles is from that of small-pox, etc. But he takes for granted the very subject to be proved, and assumes that as typhus and typhoid are different diseases, they therefore must require for their production the application of distinct specific causes.

Dr. Murchison's theories differ from those of Dr. Jenner; but the consideration of their peculiarities would occupy too much space. We may merely sum up the remarks of Dr. Bell after a number of pages devoted to the discussion of objections to them. Dr. Bell thinks that both these writers have most signally failed in their object; that their arguments consist of a series of fallacies; that their references are incorrectly quoted; and that their conclusions are untenable. Therefore, the facts which he has brought forward from the experience of Stokes and Huss, and from his own observation, will not admit of any of the explanations suggested by the theories of Drs. Jenner and Murchison, but remain as cogent teachers of the community of origin in both forms of fever.

In conclusion, he examines very briefly another argument which has been brought forward by almost every advocate of the non-identity theory, to prove that typhoid and typhus fever must arise from totally distinct causes:—

1. It is alleged that patients laboring under typhus, when admitted into a fever hospital, have been attacked with typhoid fever during their convalescence.

2. It is asserted that patients who have passed through the typhoid form have been attacked, after partial or complete recovery, with typhus. From these circumstances the conclusion is deduced that the two forms of fever must arise from different poisons, and consequently are non-identical in their origin.

A careful examination into the circumstances connected with such cases will at once show that such a conclusion is quite illogical, that the real facts connected with such instances clearly indicate a community of origin. In other words, he proves from their own premises and facts that typhoid and typhus are not dissimilar diseases, but are identical in character and origin.

---

* Diseases of Heart, p. 450.

VI.—*Case of Rheumatic Fever with Endocarditis, treated without Depletion or Mercury.* By H. H. Raymond. (Med. Times and Gazette, September 1, 1860, p. 230.)

In a case of acute rheumatism, accompanied by cardiac inflammation, Mr. Raymond remarks that he could get from the patient no history of previous disease; but the sudden and violent onset of the heart affection made him suspect it, and the suspicion in a great degree directed the treatment. It subsequently turned out that when a child she had had an acute attack of rheumatic fever. The treatment, consisting in the total absence of depleting agents, and in the use of sedatives and nourishment, was pursued solely on the merits of the case as it stood. Although the rheumatic affection was most acute, there was that about the patient which gave plain warning against bleeding and mercury, and indicated an opposite course. He thought that if the heart mischief could be checked and life supported, she would probably get well. Blisters, both over the heart and the joints, were followed by marked relief; and in each case he adopted the plan of using a number of small ones instead of one large one, as he considered them just as efficacious and less irritating. Opium produced no effect whatever, while henbane was of great service from the very first dose.

His object, he states, in publishing this case, is not to point to it as one treated on any system, as he believes that the systematic treatment of diseases is the ruin of medicine. The stimulants were by no means urgently given, but solely with the view of keeping up the action of a heart that was laboring under the difficulties of disease, which, as he rightly conjectured, were partly recent and partly of old standing. The only sound inference from this and similar cases is, not that the stimulating plan is always the right one for rheumatic fever, but that there are instances in which it is the only method of saving life.

